# Variation in the Subcellular Localization and Protein Folding Activity among *Arabidopsis thaliana* Homologs of Protein Disulfide Isomerase

**DOI:** 10.3390/biom3040848

**Published:** 2013-10-21

**Authors:** Christen Y. L. Yuen, Kristie O. Matsumoto, David A. Christopher

**Affiliations:** Department of Molecular Biosciences and Bioengineering, University of Hawaii at Manoa, Honolulu, HI 96822, USA; E-Mails: cylyuen@hawaii.edu (C.Y.L.Y.); kokazaki@hawaii.edu (K.O.M.)

**Keywords:** chaperone, endoplasmic reticulum, foldase, protein body, protein disulfide isomerase, protein folding

## Abstract

Protein disulfide isomerases (PDIs) catalyze the formation, breakage, and rearrangement of disulfide bonds to properly fold nascent polypeptides within the endoplasmic reticulum (ER). Classical animal and yeast PDIs possess two catalytic thioredoxin-like domains (*a*, *a′*) and two non-catalytic domains (*b*, *b′*), in the order *a*-*b*-*b′*-*a′*. The model plant, *Arabidopsis thaliana*, encodes 12 PDI-like proteins, six of which possess the classical PDI domain arrangement (AtPDI1 through AtPDI6). Three additional AtPDIs (AtPDI9, AtPDI10, AtPDI11) possess two thioredoxin domains, but without intervening *b-b′* domains. *C*-terminal green fluorescent protein (GFP) fusions to each of the nine dual-thioredoxin PDI homologs localized predominantly to the ER lumen when transiently expressed in protoplasts. Additionally, expression of AtPDI9:GFP-KDEL and AtPDI10: GFP-KDDL was associated with the formation of ER bodies. AtPDI9, AtPDI10, and AtPDI11 mediated the oxidative folding of alkaline phosphatase when heterologously expressed in the *Escherichia coli* protein folding mutant, *dsbA^−^*. However, only three classical AtPDIs (AtPDI2, AtPDI5, AtPDI6) functionally complemented *dsbA^−^*. Interestingly, chemical inducers of the ER unfolded protein response were previously shown to upregulate most of the AtPDIs that complemented *dsbA^−^*. The results indicate that *Arabidopsis* PDIs differ in their localization and protein folding activities to fulfill distinct molecular functions in the ER.

## 1. Introduction

Newly synthesized proteins entering the secretory pathway are folded into their native structures in the endoplasmic reticulum (ER). Protein folding within the ER typically requires the participation of two classes of accessory proteins: molecular chaperones, which bind to unfolded polypeptides and suppress their tendency to spontaneously aggregate or misfold; and foldases, which accelerate the kinetics of protein folding by catalyzing the rate-limiting steps of the folding process (reviewed in [1,2,3]). Disruption of the protein folding machinery causes misfolded proteins to accumulate within the ER, leading to a deleterious condition known as ER stress. The cellular response to ER stress is the activation of a series of signal transduction pathways collectively termed the unfolded protein response (UPR). UPR attempts to resolve ER stress through multiple mechanisms, including: (1) downregulating the expression of genes encoding secretory proteins to reduce the protein-folding load of the ER; (2) upregulating components of the ER-associated degradation (ERAD) pathway to dispose of misfolded proteins; (3) inducing genes encoding chaperones and foldases to increase the ER’s protein-folding capacity (reviewed in [4,5]).

As part of the protein folding process, many secretory and membrane proteins require the formation of intramolecular disulfide bonds between pairs of cysteine residues to stabilize their native conformation. In eukaryotes, protein disulfide isomerases (PDIs; EC 5.3.4.1) play a crucial role in this process by catalyzing the formation, breakage, and rearrangement of disulfide bonds in a wide range of client proteins [6]. The canonical *PDI* gene (*PDIA1*), first cloned and sequenced in rat, encodes a protein with four modular domains in the sequential arrangement *a-b-b'-a'*, where *a* and *a′* are catalytic domains sharing homology to thioredoxin [7], and *b* and *b′* are non-catalytic domains with tertiary structures resembling the thioredoxin fold [8]. The *a* and *a′* domains of PDIA1 possess the di-cysteine motif, CxxC, which directly participates in the thiol-disulfide exchange reactions responsible for the oxidation or reduction of disulfide bonds in substrate proteins. In addition to its function as a foldase, PDIA1 exhibits chaperone-like activity [9], and can aid the refolding of non-disulfide-bonded proteins *in vitro* [10,11]. Mammals possess an assortment of PDI-related proteins, many of which deviate from PDIA1 in terms of the number and arrangement of catalytic (*a*) and non-catalytic (*b*) domains [12]. For example, PDIA6 (also termed P5) contains two *a* domains and a single *b* domain, in the configuration *a^o^*-*a*-*b* [13]. PDIA6 exhibits disulfide oxidoreductase activity and chaperone activity *in vitro*, although both activities are lower than for PDIA1 [14,15].

In plants, PDIs have been implicated in the unfolded protein response [16], the folding of storage proteins [17,18,19] and insecticidal cyclotides [20], maturation of the embryo sac [21], and the programmed cell death of endothelial cells in developing seeds [22]. PDIs are not only found within the ER of plants, but have also been detected in vacuoles [22,23] and the nucleus [23], and in the chloroplasts of both plants [24] and unicellular green algae [25]. The plant PDI family is represented by orthologs of both PDIA1 and PDIA6, as well as novel PDI-related proteins with no obvious animal counterpart [16,26,27]. For example, the genome of the model plant, *Arabidopsis thaliana*, encodes at least 12 PDI-related proteins, including six PDIA1 orthologs (AtPDI1, AtPDI2, AtPDI3, AtPDI4, AtPDI5, AtPDI6) and two PDIA6 orthologs (AtPDI9, AtPDI10). To date, a comparative analysis of the sub-cellular locations and roles in protein folding for the various *Arabidopsis* PDI-related proteins has not been done. 

Here, we examined the cellular localization and the protein folding activity of the *Arabidopsis* PDIA1 and PDIA6 orthologs to further elucidate how the various members of the PDI family differ functionally from each other. Using fluorescent protein fusions, we demonstrated that the *Arabidopsis* PDIA6 orthologs have a subcellular localization pattern that is distinct from PDIA1 orthologs, and that overexpression of the PDIA6 orthologs in leaf protoplasts was associated with the formation of ER bodies. Furthermore, both *Arabidopsis* PDIA6 orthologs, and only three of the six PDIA1 orthologs, were able to functionally complement the *Escherichia coli* oxidative folding mutant, *dsbA^−^*. Our results provide further insight into the functional evolution of PDIs in plants.

## 2. Results and Discussion

### 2.1. Domain Architecture and Sequence Motifs of Arabidopsis PDIA1 and PDIA6 Homologs

**Figure 1:**
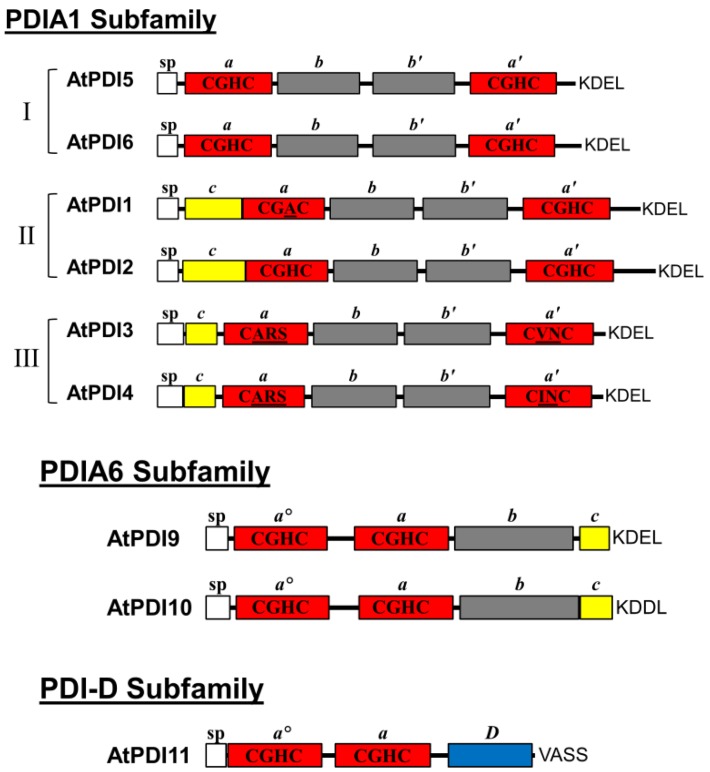
Domain arrangements of PDIA1, PDIA6, and PDI-D homologs from *Arabidopsis*. Red boxes correspond to thioredoxin-like catalytic domains (*a*, *a′*, *a^o^*), and grey boxes are non-catalytic thioredoxin-fold domains (*b, b′*). The signal peptides (sp) are represented as white boxes, and acidic regions (*c*) as yellow boxes. The *D* domain of PDI-D proteins is depicted as a blue box. The active site sequences of each catalytic domain are shown, with residues deviating from the typical CGHC motif underlined. The last four amino acids are shown at the end of each depicted protein.

In this study, we focused the analysis on the *Arabidopsis* orthologs of the mammalian PDIs, PDIA1 and PDIA6. Phylogenetic studies [16,26,37] indicate that the six PDIA1 orthologs of *Arabidopsis* form a single clade, and are divided into three evolutionary groups: I (AtPDI5 and AtPDI6), II (AtPDI1 and AtPDI2), and III (AtPDI3 and AtPDI4). All six members of the PDIA1 subfamily share the *a*-*b*-*b′*-*a′* domain configuration of *Homo sapiens* PDIA1 (HsPDIA1), and each possesses both an *N*-terminal signal peptide and the *C*-terminal ER retention motif, KDEL (Figure 1) [16,26]. However, one notable difference between HsPDIA1 and its *Arabidopsis* homologs is the presence of a short, highly acidic region (17 Asp/Glu residues over a 24-a.a. interval) located near the *C*-terminus of HsPDIA1. The acidic region of HsPDIA1, designated the *c* domain, was proposed to serve as a low-affinity, high-capacity Ca^2+^ binding domain [28]. Unlike HsPDIA1, the *Arabidopsis* orthologs of PDIA1 do not possess acidic *C*-terminal domains, although the *N*-terminal regions of the members of groups II (AtPDI1 and AtPDI2) and III (AtPDI3 and AtPDI4) are generally enriched for acidic (Asp/Glu) residues (Figure 1) [23,27], and could therefore also potentially serve as Ca^2+^ binding domains. 

The two thioredoxin domains of HsPDIA1 share the catalytic motif CGHC which is highly conserved among members of the PDI family. As shown in Table 1, the *a* and *a′* domains of AtPDI2, AtPDI5 and AtPDI6 also possess the CGHC active site sequence. On the other hand, only the *a′* domain of AtPDI1 harbors the typical CGHC motif, whereas its *a* domain contains the sequence CGAC. Interestingly, the active site sequences of group III members are highly divergent from usual CGHC motif found in PDIs. The *a* domains of AtPDI3 and AtPDI4 have the active site sequence CARS, while the *a'* domain of AtPDI3 has the sequence CVNC and, the *a'* domain of AtPDI4 has the sequence CINC. Among the *Arabidopsis* PDIA1 orthologs, only the thioredoxin domains of AtPDI3 and AtPDI4 lack a highly conserved Arg residue implicated in the re-oxidation of the active site (Table 1) [29].

**Table 1:** *Arabidopsis* homologs of PDIA1, PDIA6, and PDI-D. Active site sequence refers to the CxxC or CxxS motif of each thioredoxin domain. The position of the conserved Arg residues corresponding to R120 and R461 of HsPDIA1 were determined by CLUSTALW alignment; residues in parenthesis indicate the substitution of Arg with another residue. For the alternate names of each PDI, the names on the left are from [26], and the names on the right are from [27].

Name	AGI Number	Domain Arrangement	Active Site Sequence	Conserved Arg Positions	Alternate Names
**PDIA1**					
*Group I*					
PDI5	At1g21750	*a-b-b′-a′*	CGHC/CGHC	R127/R468	PDIL1-1/PDIL1-1
PDI6	At1g77510	*a-b-b′-a′*	CGHC/CGHC	R126/R466	PDIL1-2/PDIL1-2
*Group II*					
PDI1	At3g54960	*a-b-b′-a′*	CGAC/CGHC	R190/R532	PDIL1-3/PDIL2-1
PDI2	At5g60640	*a-b-b′-a′*	CGHC/CGHC	R194/R536	PDIL1-4/PDIL2-2
*Group III*					
PDI3	At1g52260	*a-b-b′-a′*	CARS/CVNC	(S170)/(L485)	PDIL1-5/PDIL 3-1
PDI4	At3g16110	*a-b-b′-a′*	CARS/CINC	(F168)/(S510)	PDIL1-6/PDIL3-2
**PDIA6**					
PDI9	At2g32920	*a^o^-a-b*	CGHC/CGHC	R122/R255	PDIL2-3/PDIL5-2
PDI10	At1g04980	*a^o^-a-b*	CGHC/CGHC	R124/R260	PDIL2-2/PDIL5-1
**PDI-D**					
PDI11	At2g47470	*a^o^-a-D*	CGHC/CGHC	R117/R236	PDIL2-1/PDIL4-1

*H. sapiens* PDIA6 has a structural arrangement consisting of an *N*-terminal signal peptide, two catalytic thioredoxin-like domains (*a^o^* and *a*), a single non-catalytic thioredoxin-like fold domain (*b*), a *C*-terminal acidic domain (*c*), and a KDEL ER retention signal. AtPDI9 and AtPDI10 share the same structural arrangement as HsPDIA6, except that the *C*-terminus of AtPDI10 ends with the sequence KDDL instead of KDEL (Figure 1). Although AtPDI11 possesses a tandem thioredoxin-like domain arrangement similar to that of the *N*-terminal regions of AtPDI9 and AtPDI10, the *a^o^* and *a* domains of AtPDI11 arose through a separate domain duplication event than the one responsible for the *a^o^* and *a* domains of HsPDIA6 and its *Arabidopsis* orthologs [26]. Furthermore, the *C*-terminus of AtPDI11 does not share homology to PDIA6, and instead contains the conserved *D* domain shared by members of the PDI-D subfamily [21]. The *a^o^* and *a* domains of AtPDI9, AtPDI10 and AtPDI11 all possess the common PDI active site sequence CGHC and the conserved Arg residue involved in active site re-oxidation (Table 1).

### 2.2. Fluorescent Protein Fusions of the Arabidopsis PDIA1 and PDIA6 Exhibit Distinct Localization Patterns When Transiently Expressed in Protoplasts

**Figure 2:**
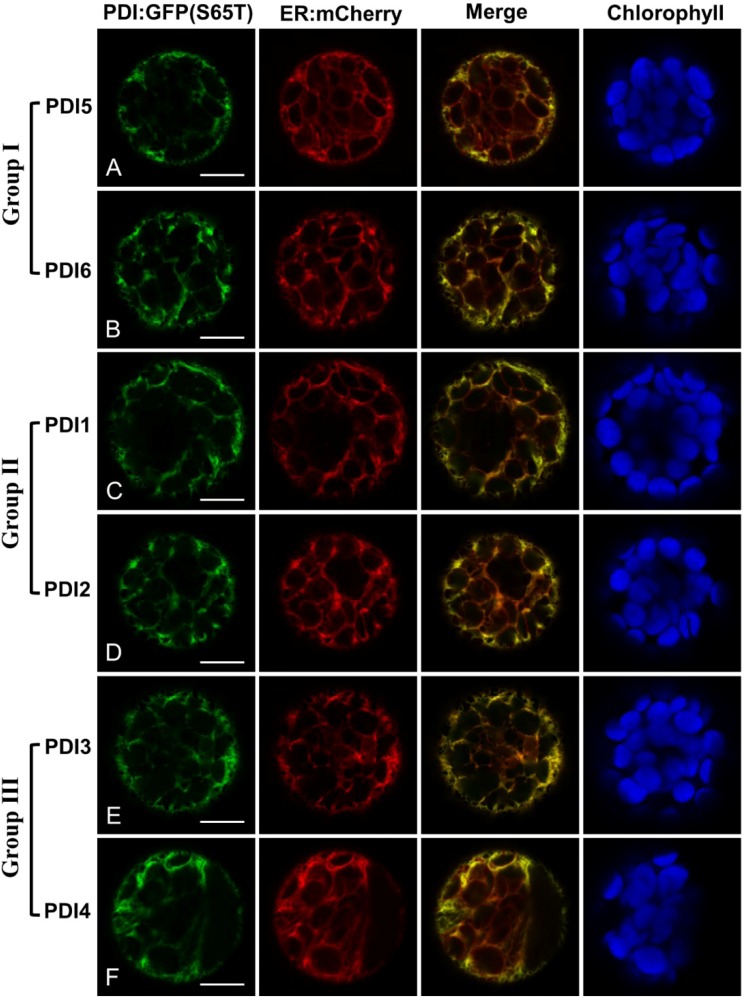
*Arabidopsis* PDIA1 homologs fused to GFP localize to the ER. (**A**) PDI5:GFP-KDEL; (**B**) PDI6:GFP-KDEL; (**C**) PDI1:GFP-KDEL; (**D**) PDI2:GFP-KDEL; (**E**) PDI3:GFP-KDEL; (**F**) PDI4:GFP-KDEL. Each chimeric fusion was co-expressed in leaf protoplasts with the ER marker, ER:mCherry. GFP signal is shown in Column 1, mCherry signal in Column 2, and a merge of both signal patterns in Column 3. Chlorophyll autofluorescence is shown in Column 4. The white bars in Column 1 represent 10 µm.

To determine the intracellular locations of the *Arabidopsis* PDIA1 homologs, constructs were developed for the expression of each of the six subfamily members fused at their *C*-termini to the green fluorescent protein variant GFP(S65T). The end of GFP(S65T) was modified in these fusions to include the KDEL motif normally present at the *C*-terminus of all six homologs (Figure 1). The fusion constructs were transiently expressed in protoplasts generated from four-week-old *Arabidopsis* rosette leaves, and their subcellular distribution analyzed by laser-scanning confocal microscopy. The localization patterns of the GFP fusion constructs were compared against ER-mCherry, a derivative of the ER marker (ER-rk) developed by Nelson *et al*. [30]. In the case of the PDIA1 group I fusions, both PDI5:GFP-KDEL (Figure 2A) and PDI6:GFP-KDEL (Figure 2B) co-localized strongly with ER-mCherry, indicating that they were predominantly retained within the ER. Similar results were obtained with the fusions corresponding to PDIA1 homologs from groups II (Figure 2C,D) and III (Figure 2E,F). 

**Figure 3:**
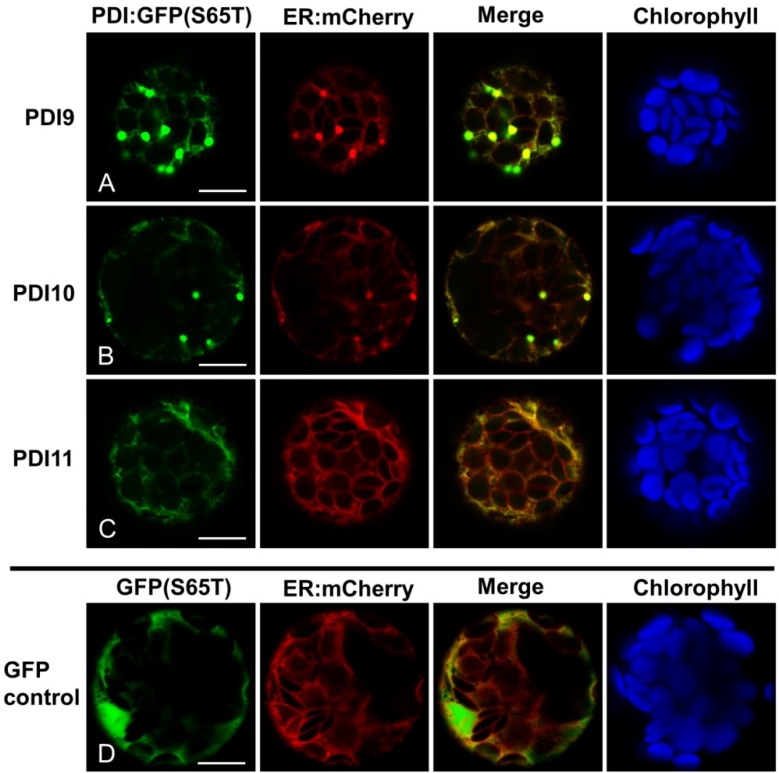
*Arabidopsis* PDIA6 and PDI-D homologs fused to GFP localize to the ER. (**A**) PDI9:GFP-KDEL; (**B**) PDI10:GFP-KDDL; (**C**) PDI11:GFP; (**D**) unfused GFP(S65T) control. Each chimeric fusion was co-expressed in leaf protoplasts with the ER marker, ER:mCherry. GFP signal is shown in Column 1, mCherry signal in Column 2, and a merge of both signal patterns in Column 3. Chlorophyll autofluorescence is shown in Column 4. The white bars in Column 1 represent 10 µm.

Translational fusions to GFP(S65T) were also generated for AtPDI9 and AtPDI10, with the *C*-terminus of GFP(S65T) modified to KDEL and KDDL, respectively. Both PDI9:GFP-KDEL and PDI10:GFP-KDDL colocalized with ER:mCherry when expressed in protoplasts (Figure 3A,B). Interestingly, the expression of PDI9:GFP-KDEL or PDI10:GFP-KDDL in protoplasts was associated with the formation of punctate structures which were strongly labeled by PDI9:GFP-KDEL and PDI10:GFP-KDDL. The punctate structures were co-labeled by the ER marker (Figure 3A,B), suggesting that they are ER-associated bodies. Since the PDI-D homolog, AtPDI11, has an *N*-terminal tandem thioredoxin domain arrangement similar to that of AtPDI9 and AtPDI10, we also examined the localization pattern of a PDI11:GFP fusion under the same experimental conditions. Despite lacking a *C*-terminal KDEL signal, we observed strong co-localization between the PDI11:GFP fusion and ER-mCherry (Figure 3C). The subcellular distribution pattern of PDI11:GFP in *Arabidopsis* protoplasts was consistent with the previously reported ER localization of a similar fusion transiently expressed in onion epidermal cells [21]. Unlike the PDI9:GFP-KDEL and PDI10:GFP-KDDL fusions, expression of AtPDI11:GFP in protoplasts was not associated with the formation of punctate structures (Figure 3C). Likewise, we did not observe ER:mCherry-labeled punctate bodies in protoplasts expressing GFP(S65T) alone (Figure 3D), or any of the PDIA1-like GFP fusions (Figure 2). Thus, the induction of ER-associated punctate bodies appeared to be specifically associated with the transient expression of the *Arabidopsis* orthologs of PDIA6. 

**Figure 4:**
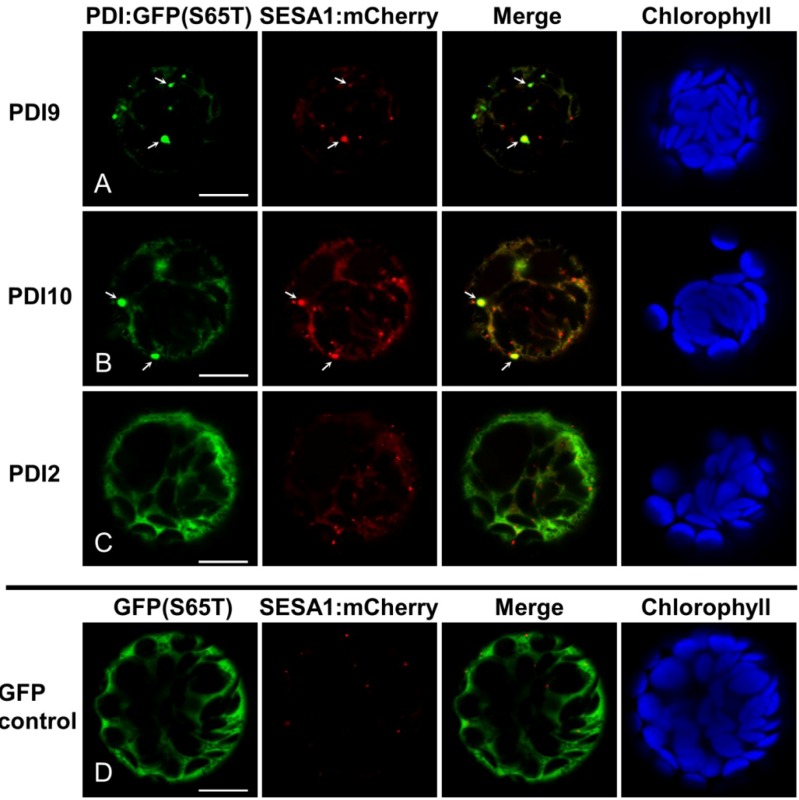
SESA1:mCherry partially localizes to punctate bodies induced by PDI9:GFP-KDEL or PDI10:GFP-KDDL expression. (**A**) PDI9:GFP-KDEL; (**B**) PDI10:GFP-KDDL; (**C**) PDI2:GFP-KDEL control (non-ER body-inducing); (**D**) unfused GFP(S56T) control. Each chimeric fusion was co-expressed in leaf protoplasts with the seed storage protein marker SESA1:mCherry. GFP signal is shown in Column 1, mCherry signal in Column 2, and a merge of both signal patterns in Column 3. Chlorophyll autofluorescence is shown in Column 4. Arrows indicate punctate bodies co-labeled by SESA1:mCherry and either (**A**) PDI9:GFP-KDEL or (**B**) PDI10:GFP-KDDL. The white bars in column 1 represent 10 µm.

To determine if the punctate labeling pattern of PDI9:GFP-KDEL and PDI10:GFP-KDDL represent storage protein-accumulating ER bodies, a seed storage protein marker was generated by fusing *Arabidopsis* seed storage albumin 1 to mCherry (SESA1:mCherry). The strong punctate signals of PDI9:GFP-KDEL and PDI10:GFP-KDDL overlapped with SESA1:mCherry fluorescence (arrows in Figure 4A,B), indicating that the punctate structures contained the seed storage protein fusion and may, therefore, be protein body-like structures within the ER. The SESA1:mCherry fusion also labeled numerous smaller punctate structures which were not co-labeled by either PDI9:GFP-KDEL or PDI10:GFP-KDDL (Figure 4A,B). These smaller punctate signals most likely correspond to pre-vacuolar compartments (PVCs) [31]. We did not detect overlap between SESA1:mCherry punctate signals and either PDI2:GFP-KDEL (which does not induce ER-associated punctate bodies; Figure 4C), or non-chimeric GFP(S65T) (Figure 4D). Our findings imply that while AtPDI9 and AtPDI10 are directed to similar ER bodies as AtSESA1, they are retained within the ER, whereas AtSESA1 leaves the ER and is trafficked to PVCs for subsequent mobilization to protein storage vacuoles.

### 2.3. Restoration of Alkaline Phosphatase Activity in the Escherichia coli Protein Folding Mutant, dsbA^−^, by a Subset of Arabidopsis PDIs

In *E. coli*, oxidative folding of alkaline phosphatase (PhoA) is mediated by the periplasmic disulfide oxidoreductase, DsbA. Loss-of-function mutations of *dsbA* disrupt alkaline phosphatase activity due to an inability of homodimeric PhoA to form two critical disulfide bonds in each of its two subunits [32]. PhoA activity can be restored to wild-type levels by the expression of recombinant human PDIA1 [33]. To determine if the *Arabidopsis* PDIA1 and PDIA6 homologs have a similar capacity to functionally complement *dsbA^−^*, their respective mature peptide coding sequences were cloned into the bacterial expression vector, pFLAG-CTS, between the vector sequences coding for the OmpA signal peptide (for periplasmic localization) and *C*-terminal FLAG tag. 

**Figure 5:**
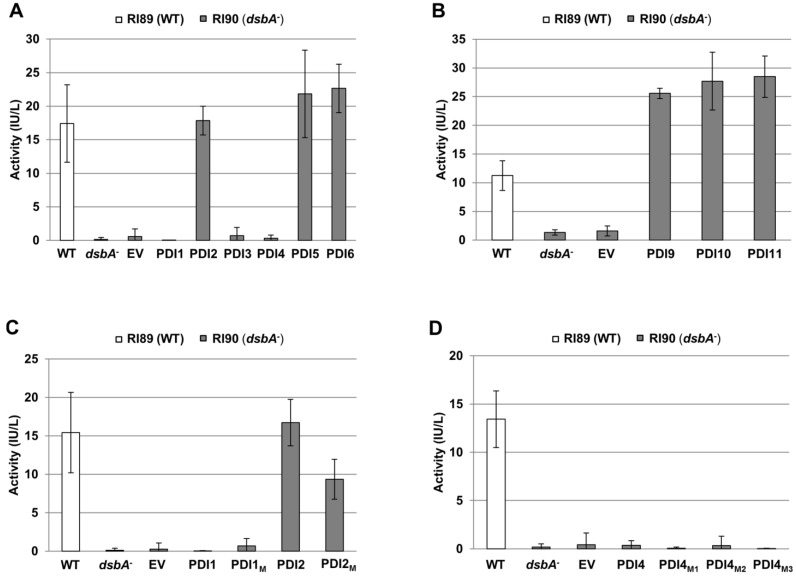
Alkaline phosphatase activity of *E. coli dsbA^−^* cells expressing *Arabidopsis* homologs of PDIA1, PDIA6, and PDI-D. Measured alkaline phosphatase activities of wild-type strain RI89 (WT), *dsbA^−^* strain RI90 (*dsbA^−^*), and RI90 cells transformed with the pFLAG-CTS empty vector (EV) or constructs heterologously expressing the following *Arabidopsis* PDIs: (**A**) PDIA1 subfamily members AtPDI1 through AtPDI6; (**B**) PDIA6 subfamily members AtPDI9 and AtPDI10, and PDI-D subfamily member PDI11; (**C**) AtPDI1, AtPDI2, and modified variants AtPDI1_M_ (*a*: CGHC) and AtPDI2_M_ (*a*: CGAC); (**D**) AtPDI4 and modified variants AtPDI4_M1_ (*a*: CARC), AtPDI4_M2_ (*a*: CGHC), and AtPDI4_M3_ (*a*: CGHC, *a′*: CGHC). Error bars represent standard deviations.

**Figure 6:**
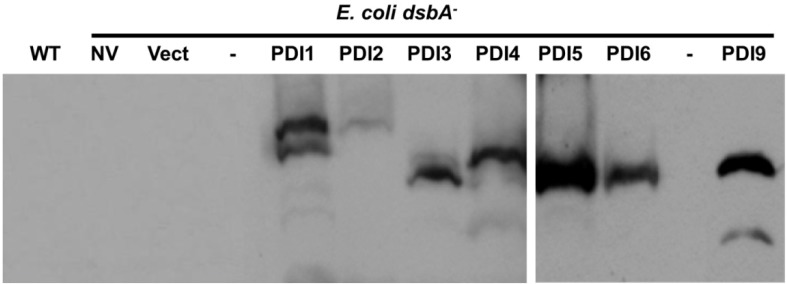
Recombinant PDI expression in the protein folding mutant *dsbA*^−^ cells. Immunoblot of proteins extracted from mutant *E. coli* strain RI90 (*dsbA*^−^), with or without constructs for the expression of recombinant PDIs. NV, no vector control; Vect, pFLAG-CTS empty vector control; PDI1, PDI2, PDI3, PDI4, PDI5 and PDI6, expression of the indicated PDI1A1 homolog; PDI9, expression of AtPDI9 (PDIA6 homolog); WT, *E. coli* strain RI89 (*dsbA*^+^) control. Lanes denoted with a dash indicate empty lanes.

The recombinant PDIs were expressed in *E. coli* strain RI90, which harbors the mutation *dsbA1::kan1*, to assess their ability to restore PhoA activity in a *dsbA^−^* null mutant background. As shown in Figure 5A, *dsbA^−^* cells transformed with the plasmids expressing recombinant AtPDI2, AtPDI5 or AtPDI6 displayed PhoA activity levels comparable to wild-type (*dsbA^+^*) strain RI89. Conversely, PhoA activity was not restored in *dsbA^−^* cells transformed with the pFLAG-CTS empty vector, or plasmids expressing recombinant AtPDI1, AtPDI3 or AtPDI4. Expression of AtPDI1, AtPDI3 and AtPDI4 in *dsbA^−^* cells was verified by immunoblot analysis (Figure 6). Interestingly, expression of PDIs with the tandem thioredoxin domain arrangement *a^o^*-*a*-*b* (AtPDI9 and AtPDI10) or *a^o^*-*a*-*D* (AtPDI11) in *dsbA^−^* cells resulted in PhoA activity levels that were greater than two-fold the activity of wild-type cells (Figure 5B), perhaps indicating that PDIs possessing a tandem thioredoxin arrangement (without the intervening *b-b′* domains) are more efficient than PDIs with the classical *a-b-b′a′* arrangement at catalyzing the oxidative folding of PhoA.

Although AtPDI1 and AtPDI2 are close paralogs, our results indicate that only the latter is capable of facilitating the oxidative folding of PhoA when heterologously expressed in *E. coli*. Since the *a* domain of AtPDI1 has an atypical catalytic motif, CGAC, we sought to address if expression of a variant of AtPDI1 in which the motif sequence was modified to CGHC (AtPDI1_M_) would restore PhoA activity in *dsbA^−^* cells, and likewise, if conversion of the CGHC motif of the *a* domain of AtPDI2 to CGAC (AtPDI2_M_) would alter its ability to mediate PhoA folding. While the level of PhoA activity in *dsbA^−^* cells expressing the AtPDI2_M_ variant was greater than cells transformed with the empty vector, it was significantly reduced relative to cells expressing unmodified AtPDI2 (Figure 5C), indicating that the sequence CGAC is less effective than the more common CGHC motif of PDIs at catalyzing disulfide bond formation. On the other hand, *dsbA^−^* cells expressing the AtPDI1_M_ variant did not display significant PhoA activity over cells transformed with either the empty vector or unmodified AtPDI1 expression construct (Figure 5C).

The active site sequences of the *a* and *a′* domains of AtPDI3 and AtPDI4 show the greatest divergence from the typical CGHC motif of PDIs. Most notably, their *a* domain active site motifs possess only a single Cys residue (CxxS instead of CxxC), which would preclude their participation in disulfide bond formation, but not isomerization [34]. Nevertheless, only a single thioredoxin domain of PDIA1 is necessary to catalyze disulfide bond formation [35]. However, even though the *a′* domains of AtPDI3 and AtPDI4 have CxxC motifs, neither protein was capable of compensating for the loss of DsbA in *E. coli* (Figure 5A). To determine if the heterologous expression of AtPDI4 harboring modifications to its *a* and *a′* catalytic motifs (normally CARS and CINC, respectively) could functionally complement the *dsbA^−^* mutation, three variant AtPDI4 constructs were generated: AtPDI4_M1_ (*a*: CARC, *a′*: CINC), AtPDI4_M2_(*a*: CGHC, *a′*: CINC), and AtPDI4_M3_(*a*: CGHC, *a′*: CGHC). The purpose of these variant constructs was to determine if the simple modification of the CARS active site sequence to CARC would enable AtPDI4 to catalyze the oxidative refolding of PhoA, or if not, if the conversion of one or both of the catalytic domain active site sequences to CGHC would be sufficient. However, none of the three modified versions of AtPDI4 restored PhoA activity in *dsbA^−^* cells (Figure 5D), suggesting that modifications outside of the active site are necessary to enable AtPDI4 to mediate PhoA refolding.

### 2.4. Functions of PDIA1 and PDIA6 Orthologs in Plants

#### 2.4.1. PDIA1-like Subfamily, Group I (AtPDI5, AtPDI6)

One mechanism by which UPR attempts to resolve ER stress is by increasing the abundance of chaperones and foldases within the ER. In mammals, PDIA1 is upregulated during ER stress as part of the UPR, and has been shown to suppress the misfolding and aggregation of a mutant form of superoxide dismutase-1 [36]. The genes encoding AtPDI5 and AtPDI6 display significant upregulation in seedlings treated with chemical inducers of ER stress [16]. Our results demonstrating that AtPDI5 and AtPDI6 can catalyze disulfide bond formation (Figure 5A) is consistent with a model whereby AtPDI5 and AtPDI6 are upregulated during UPR as part of a general strategy to increase the overall protein-folding capacity of the ER lumen.

Besides its putative function in UPR, AtPDI5 was implicated in the regulation of programmed cell death (PCD) in developing seed tissues. *AtPDI5* is expressed in the endothelial cells of developing seeds just prior to their PCD, and loss-of-function mutants of *AtPDI5* exhibit decreased seed viability [22]. A yeast two-hybrid screen for potential interactors of AtPDI5 identified three cysteine proteases, and the activity of one of these (RD21) was shown to be inhibited *in vitro* by the presence of AtPDI5 [22]. Cysteine proteases are known regulators of PCD in plants [37]. It was proposed that AtPDI5 modulates the timing of PCD of endothelial cells during embryogenesis by interacting with and chaperoning PCD-mediating cysteine proteases to suppress their activity [22]. 

AtPDI5 was demonstrated by immunoelectron microscopy to be targeted to both the ER and vacuoles of roots and developing seeds. There are several reports of the non-ER localization of PDIA1 at the cytosol, cell surface, or nucleus of animal cells [38]. However, the mechanism by which animal PDIA1 orthologs escape from the ER to go to other cellular locations remains unclear. Since AtPDI5 contains no obvious signal sequence for vacuole targeting, it was proposed that AtPDI5 is co-transported to vacuoles in developing seeds when it binds to cysteine protease, enabling it to maintain repression of cysteine protease activity outside of the ER [22]. It was further speculated that the binding of cysteine protease to AtPDI5 may masks its KDEL signal, thereby preventing the retrieval of AtPDI5 back to the ER. We did not observe evidence of vacuole labeling when PDI5:GFP-KDEL was expressed in *Arabidopsis* leaf protoplasts, which may indicate that retention within the ER represents the default state for AtPDI5 in leaves (as would be anticipated due to its *C*-terminal KDEL motif), and trafficking to other organelles only occurs when specific binding partners are expressed in other tissues, such as developing embryos. Alternatively, the *C*-terminal KDEL motif added to the end of GFP(S65T) in PDI5:GFP-KDEL may not be subjected to the proposed masking effect of interactor binding due to its distance from AtPDI5 in the fusion construct.

Recently, the rice PDIA1 group I ortholog OsPDIL1-1 was demonstrated in yeast two-hybrid assays to interact with the rice cysteine protease OsCP1, and CP43 from *Arabidopsis* [39]. The *OsPDIL1-1* T-DNA insertion mutant, *pdil1-1Δ*, displayed a shrunken seed phenotype associated with abnormal thickening of the aleurone layer, increased accumulation of several seed-associated proteins, and the formation of numerous irregular ER protein bodies (PB-I) within the endosperm [39]. In a separate study, the *OsPDIL1-1* mutants CM1787, EM44, and EM747 were found to abnormally accumulate the seed storage protein precursor proglutelin within PB-I in the ER lumen [18]. The phenotypes of these *OsPDIL1-1* mutants suggest that OsPDIL1-1 contributes to the proper folding of proglutelin and other seed storage proteins. However, it is also possible that the enhanced accumulation of seed proteins in *pdil1-1Δ* mutants is caused by the altered proteolytic activity of cysteine proteases normally regulated by OsPDIL1-1 [39].

#### 2.4.2. PDIA1-like Subfamily, Group II (AtPDI1, AtPDI2)

Since the heterologous expression of AtPDI2 can restore PhoA activity in *dsbA^−^ E. coli* cells, it is inferred that AtPDI2 has the ability to catalyze disulfide bond formation [23]. However, AtPDI1 does not display a similar ability to restore PhoA activity (Figure 5A). The *a* domain of AtPDI1 has the unusual active site motif CGAC, which lacks the third position His residue found in bacterial DsbA (CPHC) and both thioredoxin domains of eukaryotic PDI (CGHC). This His residue plays an important role in generating the oxidizing potential of the active sites of DsbA and PDI [40], and modification of the third position His to another amino acid increases the stability of DsbA in the oxidized form [41]. However, the atypical active site sequence of the AtPDI1 *a* domain does not appear to be the primary reason that AtPDI1 was unable to promote PhoA folding, as modifying the sequence to CGHC did not confer the ability to restore PhoA activity in *dsbA^−^* cells (Figure 5B).

*AtPDI1* transcript levels are upregulated during ER stress [16], suggesting that its gene product, along with AtPDI5 and AtPDI6, contribute to the UPR of *Arabidopsis*. Since AtPDI1 does not promote disulfide bond formation in PhoA, it is possible that AtPDI1 has a different enzymatic function from AtPDI5 and AtPDI6 during UPR. Alternatively, although PDIs generally appear to operate over a broad range of substrates, individual members of the mammalian PDI family display partially overlapping but distinct substrate specificities [42]. Therefore, while heterologously expressed AtPDI1 does not facilitate the formation of disulfide bonds in PhoA, we cannot exclude the possibility that it is capable of catalyzing disulfide bond formation in other substrate proteins, albeit at a lower relative efficiency than PDIs harboring two CGHC active sites. Having a narrower, or more specialized, substrate range than other *Arabidopsis* PDIs would allow AtPDI1, which has a less efficient *a* domain active site sequence, to act upon a different set of proteins during UPR than the more efficient folding catalysts AtPDI5 and AtPDI6. Although the substrate specificities of the various members of the PDI family are not well characterized, it has been shown that the *b′* domain of human PDIA1 serves as the principle peptide-binding site [43]. The *b′* domain is both necessary and sufficient for the binding of relatively small substrates, but is insufficient for the binding of large substrates, such as scrambled RNase, which require the contribution of additional domains, especially *a* and *a′*, to facilitate binding [43].

Unlike *AtPDI1*, *AtPDI2* is not induced by ER stress [16]. It is highly transcriptionally active in a variety of tissues, with prominent expression in the seed coat, siliques, and at the root tip [23]. AtPDI2 was detected by immunoelectron microscopy at the ER, the nucleus, and vacuoles [23]. When the fusion construct PDI2:GFP-KDEL was transiently expressed in protoplasts generated from the young, rapidly-expanding leaves of two-week-old *Arabidopsis* plants, in addition to labeling of the ER, ~80% of transfected protoplasts also exhibited PDI2:GFP-KDEL labeling of the nucleus [23]. However, using the same fusion construct, we failed to detect nuclear localization in leaf protoplasts generated from the older, fully-expanded leaves of four-week-old plants (Figure 2D), suggesting that the targeting of PDI2:GFP-KDEL to the nucleus is highly dependent upon the developmental stage of the protoplasts. Since AtPDI2 itself has no apparent nuclear localization signal, it was speculated that the trafficking of AtPDI2 to the nucleus may occur through a binding/co-transport mechanism similar to that proposed for the localization of AtPDI5 to vacuoles. Supporting this hypothesis, a yeast two-hybrid screen for putative interactors of AtPDI2 identified the nuclear transcription factor maternal effect embryo arrest 8 (MEE8) [23].

An *MEE8 Ds* insertion mutant displayed abnormal endosperm development and embryo arrest at the one-cell zygotic stage [44]. The ability of AtPDI2 to interact with MEE8 *in vivo*, as demonstrated by Förster Resonance Energy Transfer (FRET) analysis, coupled with its strong expression in seeds and siliques, suggested that AtPDI2 likely plays a role embryo and/or seed development [23]. However, the T-DNA knockout mutants *pdi2-1* and *pdi2-2* do not display an obvious mutant phenotype [23]. The lack of an overt phenotype for *pdi2* loss-of-function mutants may not be the result of functional redundancy with AtPDI1, because AtPDI1 and AtPDI2 have different domain active site sequences, and only AtPDI2 is able to functionally complement the *dsbA^−^* mutation in *E. coli*. Our preliminary characterization of *pdi1* loss-of-function single mutants, and *pdi1/pdi2* double mutants, has revealed no obvious phenotypes (data not shown). Thus, although PDIA1 group II orthologs are present in both monocots and dicots, and are therefore likely to possess an evolutionarily conserved function distinct from other plant PDIs, they are not strictly essential to the survival of *Arabidopsis* under laboratory conditions. In *Oldenlandia affinis*, a group II ortholog (OsPDI; GenBank accession number EF611425) was implicated in the formation of disulfide bonds in cyclotides, which are knotted circular proteins with insecticidal properties found in plants of the coffee (Rubiaceae) and violet (Violaceae) families. OsPDI interacts with the kalata B1 cyclotide precursor, Oak1, and is able to enhance the correct oxidative folding of kalata B1 *in vitro* [20].

#### 2.4.3. PDIA1-like Subfamily, Group III (AtPDI3, AtPDI4)

Of the three PDIA1-like groups in *Arabidopsis*, the members of group III show the greatest deviation from mammalian PDIA1. The *a* and *a′* domains of AtPDI3 and AtPDI4 have highly atypical active site sequences, and lack the conserved Arg residue implicated in re-oxidation of the active site (Table 1). When expressed in *E. coli*, AtPDI3 and AtPDI4 could not catalyze disulfide bond formation in PhoA, even after conversion of both active site sequences to the consensus CGHC motif of PDIs. The *AtPDI3* and *AtPDI4* genes appear to have a low basal level of expression in *Arabidopsis* seedlings, and are not induced during UPR [16]. Thus, it seems unlikely that AtPDI3 and AtPDI4 play the traditional role of PDIs as catalysts of protein folding. Notably, the *a* domains of AtPDI3 and AtPDI4 have the active site sequence CARS, and therefore lack the 2nd Cys residue of CxxC motifs. One possibility is that the single Cys residue of the CARS sequence forms a disulfide bond with the Cys residue of a client protein, leading to the formation of mixed disulfides [42]. A role in stable mixed disulfide formation, rather than substrate folding, would abrogate the need for rapid re-oxidation of the active site, which may explain the absence of the conserved Arg residues in thioredoxin domains of AtPDI3 and AtPDI4.

#### 2.4.4. PDIA6-like Subfamily (AtPDI9, AtPDI10)

**Figure 7:**
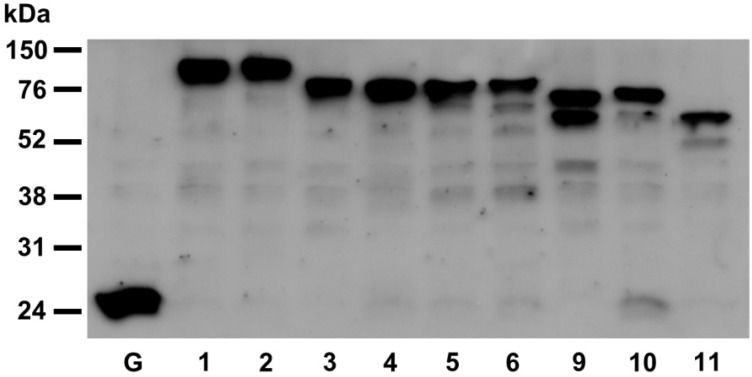
Western blot analysis of PDI-GFP fusions expressed in *Arabidopsis* leaf protoplasts. Crude protein extracts were obtained from *Arabidopsis* leaf protoplasts transfected with constructs expressing GFP(S65T) alone (control) or fused to members of the *Arabidopsis* PDI family. The proteins were immobilized on a nitrocellulose membrane and GFP was detected using a rabbit anti-GFP primary antiserum, and an anti-rabbit horseradish peroxidase conjugated secondary antiserum. G, GFP(S65T); 1, PDI1-GFP-KDEL; 2, PDI2-GFP-KDEL; 3, PDI3-GFP-KDEL; 4, PDI4-GFP-KDEL; 5, PDI5-GFP-KDEL; 6, PDI6-GFP-KDEL; PDI9-GFP-KDEL; 10, PDI10-GFP-KDDL; 11, PDI11-GFP.

The chimeric fusions, AtPDI9:GFP-KDEL and AtPDI10:GFP-KDDL, strongly labeled punctate structures when transiently expressed in leaf protoplasts. These punctate signals appear to be ER-derived protein bodies (or protein body-like structures), since they are also labeled by the ER marker, ER:mCherry, and the seed storage protein marker, SESA1:mCherry. Immunoblot analysis was used to confirm that AtPDI9:GFP-KDEL and AtPDI10:GFP-KDDL fusions were full length and the punctate bodies were not the result of aberrantly processed GFP fusion proteins (Figure 7). Since AtPDI9 and AtPDI10 are capable of catalyzing disulfide bond formation (Figure 5), the prominent localization of PDIA6 orthologs at protein bodies suggests that they may play a role in the oxidative folding of storage proteins. Indeed, the rice PDIA6 ortholog OsPDIL2-3 also localizes to protein bodies, and was shown to influence the accumulation of storage proteins in type I protein bodies (PB-I) in the ER [19]. 

The constitutive expression of a seed storage protein prolamin in transgenic rice leads to the ectopic formation of protein body-like structures in leaves and roots [45]. Our results indicate that overexpression of *Arabidopsis* PDIA6 orthologs in leaf protoplasts also induces the formation of protein body-like structures within the ER, while the overexpression of PDIA1 or PDI-D orthologs does not. This intriguing finding can serve as a useful model system to study the biogenesis of protein body formation from the ER. In *E. coli*, the molecular chaperone GroEL regulates the formation of inclusion bodies by promoting protein aggregation under certain circumstances [46]. Perhaps, AtPDI9 and AtPDI10 serve a similar function in the formation of plant protein bodies by regulating the aggregation of proteins in the ER.

## 3. Experimental

### 3.1. Constructs

The generation of constructs 35S::GFP(S65T), 35S::PDI2:GFP-KDEL, and 35S::ER-mCherry was described previously [23]. For all other fluorescent protein reporter constructs utilized in this study, plant genomic DNA sequences were amplified using primers with engineered restriction sites (Table 2). Since the genomic DNA sequence of *AtPDI1* contains an internal NcoI site, the construct 35S::PDI1:GFP-KDEL was generated by performing two separate double-digestion reactions of the AtPDI1 PCR product with NcoI/PciI and PciI/NdeI, then inserting the 0.7 kb NcoI/PciI fragment (5′-end of *AtPDI1*) and 2.6 kb PciI/NdeI fragment (3′-end of *AtPDI1*) between the NcoI and NdeI sites of 35S::PDI2:GFP-KDEL via three-way ligation. 35S::PDI3:GFP-KDEL was generated by digesting the *AtPDI3* PCR product with PciI and NdeI, and ligating between the compatible NcoI and NdeI sites of 35S::PDI2:GFP-KDEL. 35S::PDI11:GFP was created by digesting the *PDI11* PCR product with BspHI and NdeI, and ligating between the NcoI and NdeI sites of construct 35S::PDI2:GFP (no *C*-terminal KDEL modification) [23]. 

**Table 2:** List of Primers.

Construct	PCR Fragment	Forward Primer	Reverse Primer
35S::PDI1: GFP-KDEL	AtPDI1 genomic	ACAACCATGGCTTCGTCATCTACAAGTATCT	CCGACATATGCAACTCATCCTTGGAACTATCA
35S::PDI3: GFP-KDEL	AtPDI3 genomic	ATCAACATGTCGTTAATTCCAAAACCCA	TTACATATGCAATTCATCTTTAGCAGACCCA
35S::PDI4: GFP-KDEL	AtPDI4 genomic	AAACTCGAGTAACAATCATGTTGACGAAACCA	TGGCCCGGGTAACTCATCTTTACCAGACTGATC
35S::PDI5: GFP-KDEL	AtPDI5 genomic	GAACTCGAGAGCGATAATGGCGATGAG	CTCCCCGGGGAGCTCATCCTTGACTTCCT
35S::PDI6: GFP-KDEL	AtPDI6 genomic	AAACTCGAGCGATAATAATGGCGTTTAAG	AGACCCGGGCAGCTCGTCCTTTGCGGCCGT
	CaMV 35S promoter	TTCAGGGTACCTTCATGGAGTCAAAGATTCA	ATCTACTCGAGTCAAGAGTCCCCCGTG
	GFP(S65T)-KDEL:Nos 3'-UTR	CACCGACTAGTCATATGGTGAGCAAGGGCGAG	AGGATGGTCACCTAAAGCTCATCTTTGCCGTGAGTGATC
35S::PDI9: GFP-KDEL	AtPDI9 genomic	GGGCTCGAGAAAAATGTATAAATCACCATTAAC	GATCCCGGGCAACTCATCCTTAGAACCAAC
35S::PDI10: GFP-KDDL	AtPDI10 genomic	AGGCTCGAGACCATGGAAAGAAAAATGTACAAATCA	AGACATATGCAAGTCGTCCTTGGACTCAGTG
	GFP(S65T)-KDDL	CACCGACTAGTCATATGGTGAGCAAGGGCGAG	AGGATGGTCACCTAAAGaTCATCTTTGCCGT
35S::PDI11: GFP-KDEL	AtPDI11 genomic	GAAGCAGAAAAAATCATGACGAAATCTCAG	CAAGCTATATGACATATGAGAAGAAGCAAC
35S::SESA1: mCherry	AtSESA1 genomic	CACACTAGTCAAAAAATGGCAAACAAGTTG	TGAGGATCCGTAGAAAGAAGGGAATGAAG
pFLAG-PDI1	AtPDI1 cDNA	CTTCCCGGGAGAGAATGCGTCCAGTGGATC	CGAAGATCTCAACTCATCCTTGGAACTATCA
pFLAG-PDI2	AtPDI2 cDNA	TTTCCCGGGCGCTTCTTCCTCCGACGAC	TTCGTCGACCAATTCGTCCTTCGAGTCACT
pFLAG-PDI3	AtPDI3 cDNA	CATCCCGGGTTACTCATCACCCGATTCCA	TTAGTCGACCAATTCATCTTTAGCAGACCCA
pFLAG-PDI4	AtPDI4 cDNA	TGTCCCGGGCTCTGATGTCGCCGTCGAAG	TGGGTCGACTAACTCATCTTTACCAGACTGATC
pFLAG-PDI5	AtPDI5 cDNA	TATCCCGGGCGAAGAGACGGAGACGAAG	CTCAGATCTGAGCTCATCCTTGACTTCCTC
pFLAG-PDI6	AtPDI6 cDNA	TATCCCGGGCGAGGAGACGAAGGAATTTG	AGAGTCGACCAGCTCGTCCTTTGCGGCCGT
pFLAG-PDI9	AtPDI9 cDNA	CAGCCCGGGTCTTTATGGATCTTCGTCACCTG	GATgtcgacCAACTCATCCTTAGAACCAACAG
pFLAG-PDI10	AtPDI10 cDNA	GGTGGTACCCTCTATGGATCTTCGTCGCCTG	AGAGTCGACCAAGTCGTCCTTGGACTCAG
pFLAG-PDI11	AtPDI11 cDNA	AGACCCGGGTGACGATGTGGTTGTTTTGACTG	TGAGTCGACAGAAGAAGCAACGAACGTGGTTAG
pFLAG-PDI1_M_	pFLAG-PDI1(a:CGHC)	CTCCGTGGTGCGGCCACTGTCAGGCTTTGAC	GTCAAAGCCTGACAGTGGCCGCACCACGGAG
pFLAG-PDI2_M_	pFLAG-PDI2(a:CGAC)	CTCCGTGGTGTGGTGCTTGTCAGTCTCTTGC	GCAAGAGACTGACAAGCACCACACCACGGAG
pFLAG-PDI4_M1_	pFLAG-PDI4(a:CARC)	GCATTAGCTCAGCGCACCTCGCACACCAC	GTGGTGTGCGAGGTGCGCTGAGCTAATGC
pFLAG-PDI4_M2_	pFLAG-PDI4(a:CGHC)	GTTACGCGCCGTGGTGTGGTCATTGCGCTGAGCTAATGCCGAG	CTCGGCATTAGCTCAGCGCAATGACCACACCACGGCGCGTAAC
pFLAG-PDI4_M3 _	pFLAG-PDI4(a′:CGHC)	CACACACCATGGTGTGGTCATTGTGAGGCTCTGAG	CTCAGAGCCTCACAATGACCACACCATGGTGTGTG

For construction of 35S::PDI6:GFP-KDEL, a CaMV 35S promoter fragment with engineered KpnI and XhoI restriction sites was amplified from pCAMBIA1302, and inserted between the KpnI and XhoI sites of the vector pBluescript KS+. Next, a GFP(S65T)-KDEL:nopaline synthase (nos) 3'-UTR fragment with engineered SpeI and SacI sites was amplified from 35S::PDI2:GFP-KDEL, and inserted between sites SpeI and SacI. Finally, an *AtPDI6* genomic DNA fragment was amplified with engineered XhoI and XmaI sites, and inserted between the corresponding restriction sites of the intermediate plasmid to generate the final construct. To generate 35S::PDI4:GFP-KDEL and 35S::PDI5:GFP-KDEL, the genomic DNA sequences of *AtPDI4* and *AtPDI5* were amplified with flanking XhoI and XmaI sites, then ligated between the XhoI and XmaI sites of 35S::PDI6:GFP-KDEL to replace the *AtPDI6* fragment with *AtPDI4* or *AtPDI5*, respectively. Since the genomic DNA sequence of *AtPDI9* contains an internal XhoI site, construct 35S::PDI9:GFP-KDEL was created by performing two separate double-digestion reactions of *AtPDI9* with the restriction enzyme combinations XhoI/NcoI and NcoI/XmaI, and then ligating the 0.4 kb XhoI/NcoI fragment and 2.5 kb NcoI/XmaI fragment between the XhoI and XmaI sites of 35S::PDI6:GFP-KDEL via three-way ligation. To generate 35S::PDI10:GFP-KDDL, an XhoI/NdeI *AtPDI10* genomic DNA fragment and an NdeI/BstEII GFP(S65T)-KDDL fragment were simultaneously inserted between the XhoI and BstEII restriction sites of 35S::PDI5:GFP-KDEL by three-way ligation. 35S::SESA1:mCherry was created by ligating an SpeI/BamHI *AtSESA1* (*At4G27140*) fragment between the SpeI and BamHI sites of construct 35S::MEE8:mCherry [23].

Constructs for the heterologous expression of *Arabidopsis* PDIs in *E. coli* were generated in the expression vector pFLAG-CTS^TM^ (Sigma-Aldrich, St. Louis, MO, USA). The mature protein coding sequences for each PDI was amplified using primers with engineered restriction sites (Table 2). The *AtPDI1* and *AtPDI5* coding sequences were ligated between the XmaI and BglII restriction sites of pFLAG-CTS^TM^. The mature protein coding sequences for *AtPDI2*, *AtPDI3*, *AtPDI4*, *AtPDI6*, *AtPDI9* and *AtPDI11* were ligated between the XmaI and SalI sites of the vector. *AtPDI10* was ligated between the restriction sites KpnI and SalI. In the case of constructs expressing PDIs with modified versions of their active site sequences, site-directed mutagenesis was performed as described by Laible and Boonrod [47], using the primers shown in Table 2. Construct pFLAG-PDI1_M_ has the amino acid substitution A130H (*a* domain active site sequence: CGHC); pFLAG-PDI2_M_ has the substitution H134A (*a*: CGAC); pFLAG-PDI4_M1_ has the substitution S107C (*a*: CARC); and pFLAG-PDI4_M2_ has the substitutions A105G, R106H, and S107C (*a*: CGHC). The construct pFLAG-PDI4_M3_ was generated using pFLAG-PDI4_M2_ as a template to give the substitutions A105G, R106H, S107C, I447G, and N448H (*a*: CGHC, *a′*: CGHC).

### 3.2. Protoplast Transient Expression Assays

Protoplast isolation and transfection was performed essentially as described by He *et al*. [48]. The rosette leaves of four-week-old *Arabidopsis* plants were cut transversely into thin strips and incubated in 10 mL of enzyme solution (1.5% cellulase R10, 0.4% macerozyme R10, 0.4 M mannitol, 20 mM KCl, 20 mM MES, pH 5.7) for 3 h, then mixed gently with 10 mL of W5 solution (154 nM NaCl, 125 mM CaCl2, 5 mM KCl, 2 mM MES, pH 5.7), and passed through a nylon sieve to separate out non-digested leaf debris from the protoplasts in solution. The protoplasts were pelleted at 100 × *g* for 2 min, and then resuspended in fresh W5 solution to a density of 2 × 10^5^/mL, and incubated on ice for at least 30 min. The W5 solution was then removed, and the protoplasts resuspended in MMg solution (0.4 M mannitol, 15 mM MgCl_2_, 4 mM MES, pH 5.7) to a density of 2 × 10^5^/mL. The protoplasts were transfected by gently mixing 200 μL of protoplasts in MMg solution with 20 μL of plasmid DNA solution (containing ~20 μg of each construct, dissolved in water), and 220 μL of PEG solution (40% PEG, 0.2 M mannitol, 100 mM CaCl_2_). After incubating at room temperature for 15 min, the transfection step was stopped by adding 0.8 mL W5 solution. The protoplasts were spun down at 100 × *g* for 2 min, and the pelleted protoplasts were resuspended in 1 mL WI solution (0.5 M mannitol, 20 mM KCl, 4 mM MES, pH 5.7). The transfected protoplasts were incubated in the dark at room temperature for at least 18 h before being examined using an Olympus FV-1000 laser scanning confocal microscope at the Biological Electron Microscope Facility (University of Hawaii at Manoa, Honolulu, HI, USA). The excitation/emission filters utilized for fluorescence detection were 488/505–525 nm for GFP(S65T), 543/585–615 nm for mCherry, and 633/650 nm for chlorophyll autofluorescence. The GFP fusions exhibited similar expression levels in soluble protein extracts isolated from the transfected protoplasts, as determined by immunoblot analysis (Figure 6). Proteins were extracted using the EZ protein extraction method [49] by adding 100 μL buffer E and 10 μL buffer Z to the pelleted protoplasts. In Figure 7, the GFP fusions were detected by Western blot using a polyclonal rabbit anti-GFP antibody (A6455, Life Technologies, Carlsbad, CA, USA) at 1:1,000 dilution, and visualized by chemiluminescence using the Amersham ECL Western Blotting Analysis System (RPN2108, GE Healthcare, Pittsburgh, PA, USA).

### 3.3. Alkaline Phosphatase Activity Assays

*E. coli* strains RI89 (*dsbA^+^*) and RI90 (*dsbA::kan1*; RI89 genetic background) [50] were obtained from the *E. coli* Genetic Stock Center (Yale University, New Haven, CT, USA). The various PDI bacterial expression constructs and the pFLAG-CTS^TM^ empty vector were transformed into strain RI90. To measure PhoA activity, the cells were grown at 37 °C in M9 minimal media to an OD_600_ of 0.4–0.6, harvested by centrifugation, washed once with 50 mM Tris-HCl (pH 8.0), and lysed with 0.2% Triton X-100. Activity was determined using the QuantiChrom^TM^ Alkaline Phosphatase Assay Kit (BioAssay Systems, Hayward, CA, USA). Briefly, 150 µL of working solution (5 mM magnesium acetate, 10 mM p-nitrophenyl phosphate, in supplied assay buffer, pH 10.5) was added to 50 µL of lysed cells. After quickly mixing, the initial OD_405_ (t = 0) was measured for each sample, and then re-measured after 4 min (t = 4). PhoA activity (IU/L) was calculated from the OD_405_ values as described by the assay kit. The activities reported are averages derived from a minimum of 3 independent cell cultures. For Western blot analysis (Figure 6), wild-type and *dsbA^−^* cells were grown in M9 minimal media O/N at 37 °C. Cells were harvested by centrifugation, resuspended in denaturing buffer (8 M Urea, 0.1 M NaH_2_PO_4_, 0.01 M Tris-Cl, pH 8.0), and mechanically lysed using a 28.5-G needle. The protein samples were run on a 10% SDS-PAGE gel and transferred to nitrocellulose membranes. The FLAG-tagged recombinant PDIs were detected with a monoclonal mouse anti-FLAG antibody (F3165, Sigma, St. Louis, MO, USA), and visualized by chemiluminescence using the Amersham ECL Western Blotting Analysis System (Figure 6). 

## 4. Conclusions

The *Arabidopsis* genome encodes multiple homologs of the mammalian disulfide oxidoreductases PDIA1 and PDIA6. We have shown that while the members of both the PDIA1 and PDIA6 subfamilies of *Arabidopsis* localize to the ER, the PDIA6 orthologs AtPDI9 and AtPDI10 also localized strongly to protein body-like structures within the ER. These structures appeared to be induced in leaf protoplasts by the overexpression of the fusion constructs AtPDI9:GFP-KDEL and AtPDI10:GFP-KDDL, suggesting that plant PDIA6 orthologs may play a role in the biogenesis of ER bodies. Furthermore, although all six of the Arabidopsis PDIA1 orthologs possess the classical PDI domain arrangement (*a*-*b*-*b′*-*a′*), only a subset was able to facilitate the oxidative refolding of PhoA when heterologously expressed in *E. coli*. In particular, the thioredoxin domains of the PDIA1 group III paralogs AtPDI3 and AtPDI4 have highly unusual active site sequences and lack a conserved arginine residue implicated in the reoxidation of the active site, suggesting that the members of group III serve a function distinct from the typical role of PDIs in the oxidative folding of proteins. These findings highlight the functional diversity among the various members of the *Arabidopsis* PDI family.
